# Methodological Aspects of Randomized Controlled Trials for Tinnitus: A Systematic Review and How a Decision Support System Could Overcome Barriers

**DOI:** 10.3390/jcm10081737

**Published:** 2021-04-16

**Authors:** Dimitrios Kikidis, Evgenia Vassou, Winfried Schlee, Eleftheria Iliadou, Nikolaos Markatos, Aikaterini Triantafyllou, Berthold Langguth

**Affiliations:** 1 First Department of Otorhinolaryngology, Head and Neck Surgery, Hippokration General Hospital, National and Kapodistrian University of Athens, 15772 Athens, Greece; evassou@med.uoa.gr (E.V.); iliadoue@med.uoa.gr (E.I.); markatosn84@gmail.com (N.M.); katerinatriant18@gmail.com (A.T.); 2 Department of Psychiatry and Psychotherapy, Universität Regensburg, 93053 Regensburg, Germany; winfried.schlee@gmail.com (W.S.); berthold.langguth@medbo.de (B.L.)

**Keywords:** tinnitus, tinnitus treatment, randomized controlled trial

## Abstract

Although a wide range of tinnitus management interventions is currently under research and a variety of therapeutic interventions have already been applied in clinical practice, no optimal and universal tinnitus treatment has been reached yet. This fact is to some extent a consequence of the high heterogeneity of the methodologies used in tinnitus related clinical studies. In this manuscript, we have identified, summarized, and critically appraised tinnitus-related randomized clinical trials since 2010, aiming at systematically mapping the research conducted in this area. The results of our analysis of the 73 included randomized clinical trials provide important insight on the identification of limitations of previous works, methodological pitfalls or gaps in current knowledge, a prerequisite for the adequate interpretation of current literature and execution of future studies.

## 1. Introduction

Tinnitus is traditionally described as the perception of a sound in the absence of corresponding external stimuli [1]. In a very recent consensus article, a more precise definition of tinnitus has been proposed: Tinnitus was defined as “the conscious awareness of a tonal and/or noise sound for which there is no identifiable corresponding external acoustic source” and tinnitus disorder was defined as “tinnitus plus tinnitus-associated emotional distress and functional disability” [2]. Tinnitus is considered an enigmatic situation and universally accepted answers to fundamental questions about its pathophysiology, course, and optimal treatment are still pending [1,3,4]. Its prevalence is estimated more than 10% in the general population; however, it is considered bothersome only in approximately 1% [3,4]. These numbers are of paramount importance, since, according to the recently released European Tinnitus Guidelines, clinical approach and decision ma-king should take into account not only tinnitus existence but also patient’s reaction to tinnitus [1]. Tinnitus is considered as a symptom well tolerated by the majority of individuals; however, it might cause levels of annoyance which can be adequate to make tinnitus the determining factor for significant impairment of the perceived health status and the overall quality of life.

One of the few things considered common ground among tinnitus community is that no optimal and universal tinnitus treatment has been reached yet [1,5]. Despite the fact that a wide range of interventions including, but not limited to, drugs and medicinal products, sound amplification, sound therapy, psychological interventions, and transcranial magnetic stimulation have been applied, none of them is universally accepted as an adequate and globally effective solution for the whole spectrum of tinnitus sufferers [1].

Hence, there is a pattern across the tinnitus literature according to which, a varying subgroup of responders is found in most of the studies [5]. This could be attributed to statistical variance, but it could also be claimed that some therapeutic interventions could potentially be beneficial in a specific subgroup of patients with identifiable characteristics. Very few studies, however, attempt to create and identify a certain profile correlating with treatment response and the main research question is limited to whether an intervention is effective or not in a group of patients, rather than which factors influence treatment response [6,7].

Moreover, tinnitus related literature has some specific barriers, on top of the issues identified as problematic in medical literature in general, such as sample size calculation, study settings, statistical analysis, and selection bias [8]. These drawbacks include the heterogeneity of tinnitus patients, the fluctuation in tinnitus perception, the subjective nature of tinnitus and therefore the lack of objective outcome measures, the common existence of comorbidities, as well as their interaction with tinnitus perception and the different perception of tinnitus in different cultures, as well as in different times by the same individual [9,10,11].

Consequently, it could be stated that the reasons for the lack of an established and effective treatment are both native and intrinsic, as well as subjective. Aim of this paper is to summarize factors, objective restraints, methodological flaws, and research insufficiencies, in order to provide some explanation for the fact that no universal tinnitus treatment has been established yet.

A systematic review of the tinnitus literature including Randomized Clinical Trial (RCTs) has been conducted aiming towards the identification of common methodological flaws and insufficiencies. Finally, a brief overview of a Decision Support System (DSS) which is the core outcome of EU-funded project Unification of Treatments and Interventions for Tinnitus Patients (UNITI) is presented [12]. The DSS takes into account epidemiological data, audiological measurements, genetic background and socioeconomic data in order to choose the optimal treatment out of the most widely used treatments (sound therapy, sound amplification, Cognitive Behavioral Therapy and Structured Counselling) at an individualized level [12].

## 2. Materials and Methods

This paper is a systematic review of literature aiming at the extraction of the characteristics of the RCTs published from 2010 to 2020 in the field of tinnitus, drafted according to PRISMA guidelines [13].

The main goal of this paper is to map and evaluate the methodology of RCTs with regards to inclusion criteria, outcome measures, consideration of confounding factors like hearing loss and whether special care was taken in order to minimize the impact of tinnitus specific confounders such as fluctuations over time or unstable treatment response. This information may lead to useful conclusions about systematic bias, methodological flaws, drawbacks, and gaps in the body of RCTs targeting tinnitus treatment.

Review question was set as following: Which were the inclusion criteria: age, characteristics of tinnitus (primary complaint, laterality, chronic/acute, onset, intermittent cha-racter, etc.), procedure, outcome measures used, study methods (sample size, power analysis, randomization, etc.), and study timeline—duration of the RCTs published between 2010 and 2020. Additionally, the research aims were formulated according to the PICO template as following:

People: Adults with history of tinnitus.

Intervention: Any kind of intervention aiming towards tinnitus treatment, care and compensation.

Comparison: Non applicable.

Outcome: Methodological aspects of tinnitus RCTs (inclusion criteria, study procedures, study outcome measures, study timeline).

### 2.1. Inclusion Criteria

Target population consists of adults with tinnitus. Trials targeting pulsatile tinnitus were excluded. Only RCTs published between 30 June 2010 and 1 July 2020, with at least 30 participants were included. The purpose of setting these inclusion criteria was to critically examine the methodological adequacy and patterns of barriers and limitations occurring in tinnitus literature in its highest level according to their type and level of evidence provided.

### 2.2. Exclusion Criteria

Protocols of RCTs, as well as RCTs mainly targeting entities other than tinnitus, even if tinnitus could occur as symptom in their context (e.g., sudden hearing loss, Meniere disease) were excluded. Any type of study other than RCT or studies written in a language other than English were excluded.

### 2.3. Information Sources 

Information sources included 3 major databases (Medline, Central, and Scopus). All searches were conducted by two authors independently. The results were then hand-searched [14].

### 2.4. Search

The search syntax used in Medline was broad: “tinnitus AND random*”. Similar approach was used in the other two databases. Filters applied were “English language”, “10 years” and “Randomized Controlled Trial”.

### 2.5. Study Selection

Studies obtained from the aforementioned search have been reviewed independently by two authors. In that stage of analysis, the authors have identified duplicates or multiple reports of the same study, by first examining the titles and abstracts of the yielded studies and then their full text. Results of grey literature, trial registration platforms, and conferences were then added. The senior author resolved any disagreements.

### 2.6. Data Collection Process

Two reviewers have screened full-text articles and produced a matrix of relevant data independently [15]. Any ambiguities on data charting would be discussed and resolved by the senior author.

### 2.7. Extracted Data Items

- Study identification
  Author, year of publication.
- Methods and inclusion criteria

Population (sample size, whether specific age range was stated and if yes, the actual age range). Existence of an inclusion criterion determining a minimum tinnitus duration reported by the patients or not.

Existence of an inclusion criterion determining whether patients reported tinnitus as a primary complain or not.

Consideration of hearing loss both in inclusion criteria (range and thresholds of hearing loss provided) as well as in data analysis and interpretation.

Whether a limitation in regards to tinnitus laterality was present or not (whether only unilateral or bilateral cases were included, whether both unilateral and bilateral were included or whether no relevant criterion was set).

- Methods and study procedures

The primary objective of the study.

The treatments applied in the intervention and the control group.

Whether randomization procedure was properly and adequately described. Metric for this was the ability to reproduce the procedure based on the details provided.

Whether power analysis was presented (either ad hoc or post hoc) and the estimated power if available.

Follow up period duration and schedule of follow up visits.

- Methods and outcome measures

Outcome measures used, and whether a primary outcome measure was clearly defined.

- Results

Results in terms both of clinically and statistically significant improvement in each one of the groups as well as of difference between groups. An estimation of whether intervention was considered effective is provided, based on whether interventional group showed statistical and clinical improvement in the primary outcome measure compared to baseline and also a statistically significant difference was found, compared to the control arm, in the case no intervention is conducted (no placebo, sham, or waiting list).

### 2.8. Risk of Bias in Individual Studies

In view of the nature of our research question and the objectives of this systematic review, potential bias and methodological pitfalls of individual studies make part of the core analysis and results interpretation.

### 2.9. Synthesis of Results

We present the included studies and summarize the extracted data items in form of tables, with emphasis on our predefined scientific queries. Further analysis in plain text and interpretation of the results shall be found in the corresponding discussion sections.

## 3. Results

### 3.1. Study Selection

**Figure 1:**
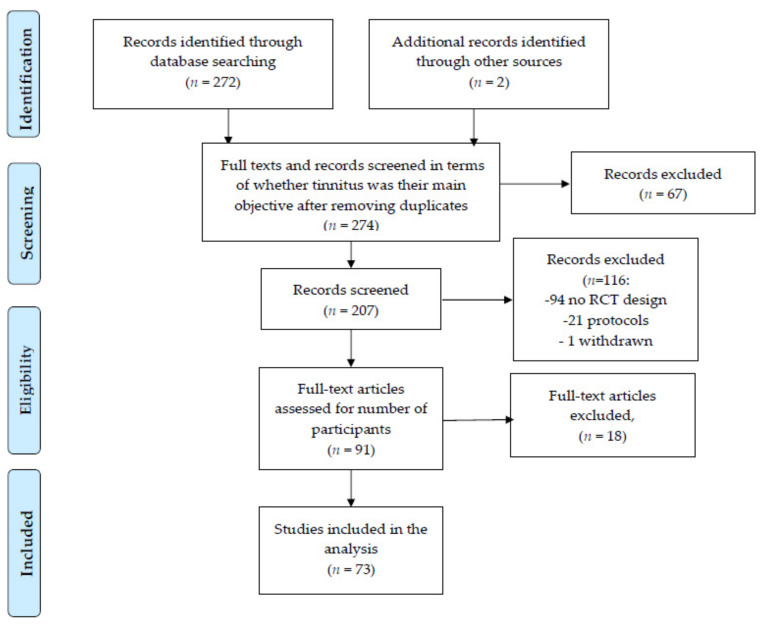
Study selection PRISMA flow diagram.

Seventy-three articles were identified. The screening procedure is reflected in Figure 1.

### 3.2. Study Characteristics

A detailed summary of the characteristics of the eligible studies can be found in the Table 1 and Table 2.

### 3.3. Results of Individuals Studies

#### 3.3.1. Methods and Inclusion Criteria

Across studies, participants’ screening and recruitment are focusing on participants’ age and presence of tinnitus, while only a small proportion of studies use specific characteristics of tinnitus as inclusion criteria (Table 1). With regards to age, 20 studies set as participants’ age upper limit the seventh decade of life, while 27 of them do not provide a clear report of this information. Seven studies have excluded participants for whom tinnitus was not their primary complaint, while there is a large heterogeneity among studies with regards to the tinnitus onset and minimum duration criterion; 2, 3, 6, and 12 months have all been used in the studies included in this review. In sixteen of them, no specification on time and/or duration is provided. Whether tinnitus is continuous or intermittent was not specified as an inclusion criterion in the investigated studies. Many studies defined a minimum annoyance level as inclusion criterion, measured either by means of simple visual analog scales (VAS) or through specific questionnaires (Tinnitus Functional Index (TFI), Tinnitus Handicap Index (THI), Tinnitus Handicap Questionnaire (THQ), and the Tinnitus Acceptance Questionnaire (TAQ)). Four studies excluded participants with hearing loss, while the grand majority of studies do not mention the hearing status of their sample. No study involving other than hearing aid fitting as study intervention had screened candidates for the use of hearing aids prior to their entry to the study.

#### 3.3.2. Methods and Study Procedures

Median number of participants per RCT was as low as 54, whereas 11 of the included studies had a sample size of more than 100 subjects. The vast majority of the studies (46 out of 73) did not provide power analysis nor details on the sample size calculation prior to the study execution.

Across studies, randomization procedure was not described in a clear manner in 46 of the studies (62.16%) (absence of specific methodology, inadequacy of the reported method to the study design).

Primary objective of the included RCTs is the evaluation of specific therapeutic approaches; seventeen of them focus on transcranial magnetic stimulation (TMS) [28,33,41,42,44,45,48,54,67,71,72,76,79,82,85,89,90,91], four on acupuncture as monotherapy [25,51,53,66], more than ten on cognitive behavioral treatment, relaxation and mindfulness [20,21,23,26,35,40,47,61,65,68,69,70,75,81,92], seven on provision of hearing aids [38,41,63,84,87,93,94], 8 on sound therapy [24,39,55,60,86,95,96,97], two on electrical stimulation [29,73,88], while more than twenty evaluate the efficacy of various pharmacological interventions [18,19,22,31,36,37,50,56,57,58,59,64,74,77,78,80,83,84,93] and one the efficacy of the Acoustic CR neuromodulation device [30].

The duration of participants’ follow-up ranged from immediate after treatment up to 26 months [18,98].

#### 3.3.3. Methods and Outcome Measures

Outcome measures used across studies vary and range from simplistic VAS to specialized and cross-culturally validated questionnaires; 14 studies have used the TFI; 9 the THI; 9 the THQ; 2 the TAQ. The majority of studies use more than one questionnaire, covering tinnitus and its comorbidities (e.g., the Hospital Anxiety and Depression Scale or HADS and the Patient Health Questionnaire—Depression or PHQ-D) (for more details, see Table 2).

## 4. Discussion

### 4.1. Tinnitus Duration and Intermittent Character

Tinnitus is a subjective symptom and in many cases it fluctuates over time [1]. Typically, patients report either fluctuations that might or might not be influenced by external factors or by their emotional status, for example levels of environmental noise or stress [1]. In the vast majority of studies, the outcome measures consist of questionnaires that are handed out at specific time points and supposed to evaluate a certain period of time [6]. This method has a fundamental flaw by default: even if patients are asked to provide information about the tinnitus severity over a defined time period (e.g., one week), the tinnitus severity at the moment when the questionnaire is filled out dominates. Tinnitus fluctuations over time or even periods without tinnitus are typically not sufficiently reflected due to memory and reporting bias. When patients are asked to fill in the questionnaire, the results will depend on their emotional status in general and particularly at the time information was provided, their overall attitude and tinnitus perception, and also on their tendency to focus on negative aspects. These confounding factors influence both the presence as well as the level of annoyance and consequently the tinnitus reporting. Therefore, they can be considered as an intrinsic difficulty that is present as a systematic bias across tinnitus related studies.

A potential solution to this could be the use of ecological momentary assessment, which is commonly integrated through mobile applications and allows ongoing recording of fluctuations in tinnitus severity as well as the correlation with certain incidents and behaviors which are captured at the same time (e.g., environmental noise, road traffic, etc.) [12]. This approach, if not well designed or capable of adjustments, may contradict efforts of habituation, since it requires that patients be frequently occupied with their tinnitus and its characteristics.

Only one of the studies took into account the duration of tinnitus within specific time intervals [80]. This finding is remarkable, as an expert consensus initiative from 2007 for tinnitus assessment and outcome measurement proposed that tinnitus patients should be asked about which percentage of their time they perceive their tinnitus?” [99].

Of course, even if asked, this information would be difficult to collect, due to recall bias and inability of patients to provide reliable information in regards to tinnitus duration due to different factors commonly mentioned, including fluctuation of tinnitus occurrence and perceived loudness if present, masking in noisy environments and lack of focus. Intrinsic issues already mentioned have not allowed a universally accepted answer to fundamental question in regards to tinnitus fluctuations, like whether patients with intermittent tinnitus tend to experience less annoyance or whether their treatment response is expected to be better [100]. Consequently, tinnitus duration and fluctuation and whether it was intermittent or not and under which conditions, was not taken into account as a factor in data analysis and interpretation neither, which is a possibly interesting point that should be considered by future studies.

### 4.2. Level of Perceived Annoyance

One of the few things that are considered common ground in tinnitus literature is the fact that the majority of people with tinnitus do not consider their tinnitus bothersome [4]. Overall prevalence, often replicated in the introductory parts of tinnitus studies considered as exceeding 10% in the general population is based on surveys in large samples [4]. On one hand, in the generation of these epidemiological data, some methodological considerations might arise about the criterion used to define tinnitus. On the other hand, the phrasing of the probably largest survey (“In the past 12 months, have you been bothered by ringing, roaring, or buzzing in your ears or head that lasts for 5 min or more?) seems clear enough in terms of adequate duration (excluding brief spikes), type of sound (noise rather than hallucinations), and time frame (one year and not whole life time). In any case, reproducibility of similar numbers in different countries confirms that these estimations should be close to reality [101].

As expected, the number of tinnitus sufferers seeking help by health professionals are much lower than the estimated prevalence [1]. This is of course easily explained by the fact that tinnitus is considered either as not a problem, or a small problem, often reported as non-bothersome. This also reflects to common clinical experience, according to which, a considerable group of patients with other chief complaints might mention tinnitus only when specifically asked. At the same time there is a subgroup of people with catastrophic tinnitus, who describe their tinnitus and the consequences as dramatical.

The discrepancy between the number of people with tinnitus and the number of those who seek medical help might also partly be due to the limited therapeutic options. A person suffering from tinnitus, who is told by the physician that there are no established possibilities to reduce the loudness of the tinnitus, might try to accept the situation without seeking further medical help.

As a result, it would be expected that tinnitus treatment studies, aiming to offer a solution to tinnitus sufferers, should select their participants accordingly and only include patients with a satisfactory level of annoyance, in order to fulfill a fundamental principle of medical research: ability to replicate their results to the target population.

According to the findings of our review, only 22 out of 73 RCTs clearly mention a minimum level of tinnitus annoyance in their inclusion criteria (Table 1). All popular questionnaires (THI, TFI, and TQ) are used, as well as Visual Analog Scales. THI is used in 12 of the studies, thresholds for inclusion however vary from 18 to 38 with four additional intermittent values: 20, 25, and 30. Only one study has set both lower and upper limits, using TFI, in order to define a certain range [80].

Of course, it is reasonable to assume that individuals with non-bothersome tinnitus will not easily reach a tinnitus clinic and on top of this, be motivated for a usually demanding participation in an RCT. In addition, baseline values give an estimation of the overall annoyance.

Including a reasonable level of annoyance, using the outcome measures chosen for the specific RCT ideally not only setting lower but also upper limits, should be considered good practice in future RCTs.

### 4.3. Tinnitus Audiological Characteristics

Tinnitus frequency can vary from constant to less than weekly. There is also a considerable proportion of patients who state that their tinnitus is only detectable in the absence of any acoustic stimulation, typically before they fall asleep. On top of this, there is a wide range of sounds considered similar to the type of tinnitus sound. Typically, tinnitus pitch is better matched with high frequencies, although there is a considerable proportion of patients who either cannot easily identify a matching sound or better attribute to low frequencies [101]. Determination of tinnitus pitch, loudness, and minimum masking level can be useful in clinical practice, in spite of their questionable role and their fluctuating nature.

However, a robust relationship between tinnitus pitch, loudness and masking level and tinnitus prognosis and severity in terms of annoyance, functionality and handicap has not been established [102]. This means that in the studies investigating the effect of various treatments these characteristics are not useful as outcome parameter. This has been confirmed in our review, in which none of the studies used this type of data neither for outcome measurement, nor as a predictor for treatment outcome.

### 4.4. Tinnitus and Hearing Loss

It is widely reproduced in the literature that hearing loss is present in approximately 90% of tinnitus patients [4]. However, it is also common ground that existence as well as degree of hearing loss are not able to predict tinnitus occurrence and severity [1]. Since pathophysiology of tinnitus is complex and involves both auditory and brain function, it is impressive that hearing loss, although present in the vast majority of tinnitus patients, has not been thoroughly studied as a prognostic factor of tinnitus course, prognosis, and treatment response [1].

This gap is clearly reflected in the extracted literature. Only seven out of 73 studies clearly state in their inclusion criteria that tinnitus was considered as primary complaint by the participants (Table 1). It could be assumed that patients with hearing loss as their primary complaint would not be motivated to participate in tinnitus oriented RCTs. This means that an unknown proportion of study participants could have tinnitus, but not as primary complaint. The primary complaint could be hearing loss and tinnitus only the secondary complaint. Moreover, it is commonly seen in clinical practice that patients present with their primary complaint of tinnitus, but when they are clinically evaluated it is discovered that their main complaint and everyday handicap is their hearing loss. Consequently, and in accordance with common clinical experience, it could be hypothesized that these groups of patients are not homogenous in principle and combine patients in a wide spectrum between hearing loss and tinnitus as primary complaints—and all the shades in between. It is assumed that this could influence results and treatment response, especially in treatments like hearing aids. On top of this, even if identifying tinnitus as primary (but not only) complaint could potentially improve sample homogeneity, it could still exclude a significant group of patients who would consider hearing loss as their cardinal problem and also have adequately bothersome or even catastrophic tinnitus at the same time.

If just dealing with the existence of hearing loss is complex, taking the degree of hearing loss into account is even more challenging. More than one third of RCTs (25 out of 73) include a range of hearing loss in their inclusion criteria, whereas none of them analyzed the audiogram as a predictor for treatment response. Even those studies in which hearing levels were mentioned as inclusion criterion, they have typically vague descriptions of hearing functions, such as “normal hearing levels”, hearing levels allowing conversation or mild, moderate, or severe hearing loss without an explicit definition or the respective thresholds.

In accordance, among the studies that evaluated other interventions than hearing aids, there was no study that took into consideration the use of hearing aids neither as an inclusion/exclusion criterion nor as a predictor nor as a confounder. The latter is potentially a hidden but significant risk of bias, since tinnitus improvement is considered as high as 55% in several case series analyzing hearing aids [5]. This could influence results in two ways; first, a selection bias, since only patients were included in the trial, in which hearing aid use was not effective to sufficiently decrease tinnitus annoyance; second, an unclear effect of a prolonged or recent use of hearing aids, which might influence the performance of an unknown proportion of hearing aids users both in the interventional and control arms. A clear exclusion of patients with a relatively recent hearing aid fitting should be considered as good practice in future RCTs.

The currently starting UNITI trial is strategically planned as an attempt to overcome the mentioned issues. Only patients with tinnitus as primary complaint will participate, and degree of hearing loss will be analyzed with sophisticated techniques as a potential confounder for treatment response. In addition, the efficacy of hearing aids as a sole measure to improve tinnitus will be tested for the first time in the context of a RCT against interventions based on other disciplines, like CBT [12].

### 4.5. Remarks on Study Methodology

Tinnitus interventions, in accordance with tinnitus pathophysiology, are heterogenous. In the RCTs collected, a wide spectrum of therapeutic strategies is performed ranging from transcranial magnetic and vagus nerve stimulation to internet-based CBT and altered/notched music (Table 2). A pattern that causes deviation from an optimal study design and is valid for various interventions is the inability to blind patients with respect to control interventions. For example, a blinded RCT comparing true and sham hearing aids is not feasible, since participants in the sham group will immediately recognize the sham devices, given that they will be unable to provide acoustic amplification.

Apart from intrinsic limitations and barriers, tinnitus literature also suffers from methodological insufficiencies which are common in other fields, as well. Median number of participants per RCT is as low as 54, whereas only 10 exceed 100 participants. Most of the studies (46 out of 73) did not provide power analysis; hence, the rest reported power over 80%. The same proportion of papers did not provide a clear, detailed, and reproducible description of their randomization procedure, a fact that clearly questions their qua-lity. Moreover, randomization procedure was found to be unclear in 46 of the studies (63.01%) under the sense, that relevant information provided was generic and not adequate for the procedure to be replicated.

Although out of the scope of this review, what needs to be underlined is that many of the RCTs concerning tinnitus are supported by pharmaceutical companies or hearing and tinnitus-related devices manufacturers. So, results must always be read with caution and extra consideration for potential biases or conflicts of interests.

### 4.6. Outcome Measures

Tinnitus is a condition affecting everyday life in many ways, causing a list of issues including, but not limited to annoyance, functional disturbances, tinnitus intrusiveness and acceptance, disability for certain actions and tasks like concentration, ability to ignore negative emotions, sense of control, malaise, and loathsomeness. Hall et al. (2019) have recently suggested proper outcome measures for each type of intervention. These recommendations could not have been applied to the body of the RCTs examined; however, lack of justification for the choice of a certain outcome measure is the rule [103].

Majority of studies (63 out of 73, 86.30%) use more than one outcome measures, a procedure that has been proposed to enable comparison across trials [99,104]. However, the use of multiple outcome measurements requirerequires the a priori definition of the primary outcome, which was the case only in 14 studies out of the 64. It is also interesting, that 34 of the RCTs use at least three outcome measures, which shows a relatively wide range of domains targeted and also increases the possibility of results ought to randomness.

### 4.7. Time Course

Relatively little is known about the course of tinnitus over time [7,105,106,107]. There are a lot of factors contributing to this. Tinnitus installation is often prolonged and there is a considerable proportion of patients that cannot clearly identify an exact date of tinnitus onset. Especially in the cases where tinnitus habituation has occurred, patients may not clearly recall or may underestimate both time of onset or severity of their tinnitus, when they are asked about it or when they are filling retrospectively a relevant questionnaire. A considerable proportion with total relapse might not even contact health services, and therefore never be recorded, which means that estimation of the course of tinnitus over time is in current studies at based assessed retrospectively by questionnaires, which are subject to recall bias as well as to suboptimal phrasing of the relevant questions in the self-filled questionnaires. Consequently, there is lack of reliable information about the actual incidence, the course and the profile of the patients who experience tinnitus for a short period of time and then stop experiencing it. On top of this, patient trajectories differ strongly across countries depending in the health system.

Tinnitus is usually dichotomized into acute and chronic; however, recent European guidelines have also included the sub-acute type (from 3 to 6 months), in order to reflect the transition from acute to chronic tinnitus [1]. However, all these definitions are arbitrary, and little is known about differences in the pathophysiology of acute and chronic tinnitus and the time when this transition occurs. It is remarkable that only two of the RCTs identified focused on acute tinnitus [50,74].

The majority of the studies (58 of 73) clearly defines a minimum time interval from tinnitus onset, however variability in time intervals is large. Eleven trials set as minimum duration 3 months and 22 the 6 months interval, whereas a wide range of smaller or larger intervals occur. This variance may be relevant for the tinnitus course, since a recent systematic review has indicated a statistically significant decrease in the impact of tinnitus over time, although clinical significance could not be interpreted due to heterogeneity [108]. This practically means that in the comparison between RCTs differences in the tinnitus duration might matter.

### 4.8. Trial Design and Results

As expected, there is a large heterogeneity among the RCTs included. About one third of the included studies examine the efficacy of pharmaceutical agents either as sy-stemic or as topical administration. Second most common topic is various types of TMS, whereas 10 focus on CBT either face to face or online, 5 on non-CBT psychological interventions, 7 on HAs (alone or in combination with motivational interview), and 8 on sound therapy. Finally, acupuncture and laser beam have also been evaluated as monotherapy for chronic tinnitus [25,27,34,43,51,53,66]. The variance of the interventions with respect to their intended mechanisms, targets and duration should have led to different trial designs in terms of outcome measures as well as follow up schedule. For instance, TMS is usually implemented in strict and well-defined time periods (typically one to two weeks), whereas CBT is an intervention lasting several weeks and should be finished before the effect can be evaluated. At the same time, HAs have a continuous and possibly long-lasting effect. This is not reflected in the design of the studies, since criteria in regards to tinnitus onset, follow up duration and outcome measures are more or less equally distributed in these sub-groups of RCTs.

It is noteworthy that two third of control groups use different types of methods in order to be non-interventional: placebo, sham devices or interventions, participants from the waiting list or usual care. Ideally, the recommendation is to use best available treatment instead of placebo, at least for pharmacological studies. This is probably not applicable in the tinnitus field, since universally acceptable treatment is pending [109]. Hence, majority of the remaining studies use as control arms active interventions of the same discipline (different TMS protocols, HA fitting parameters, stimuli used for sound therapies). Very few RCTs compare two totally independent interventions. This design should be considered in future studies, because on top of efficacy superiority, it could potentially identify profiles of patients who could be more prompt to one intervention compared to other.

Although evaluation of results was not within the core scopes of this review, it should be mentioned, that roughly one third of the RCTs concluded that the intervention tested was considered effective. RCTs targeting CBT and different types of sound therapy re-presented more than one third of the RCTs with a positive outcome, whereas their proportion in the whole body of RCTs was significantly lower (13.6% and 10.96%, respectively). All other types of interventions had at least one clinical trial with a positive result (superiority against the control intervention).

One important aspect is that with regards to several RCTs in which different active interventions were compared, it remains unclear whether the results differ from placebo. Even if there were significant within arm comparisons for all investigated interventions, one cannot unambiguously differentiate between spontaneous improvement and an effect of the investigated intervention.

Moreover, with very few exceptions, only statistical and not clinical significance was examined, and any minimum benefit considered as significant was set ad hoc.

## 5. Conclusions

Tinnitus is a clinical enigma for many reasons, most of which are intrinsic. RCTs in tinnitus field suffer from many methodological flaws, including lack of strict and well-defined inclusion criteria, failure to eliminate confounders like hearing loss, stress, and tinnitus duration, variance in outcome measures and interventions, and barriers in design. Future RCTs should clearly set a minimum level of tinnitus related annoyance, range of age and tinnitus duration, hearing ability, as inclusion criteria, choose appropriate outcome measures for the intervention tested and interpret clinical on top of statistical signi-ficance.

Design of future trials should take into account issues, barriers and problems identified in this review. Patients with intermittent tinnitus should probably be included in different studies than those with tinnitus lasting all day. Although tinnitus onset might not be always clear, the majority of studies seem targeting chronic tinnitus (>6 months) and a gap of studies targeting acute tinnitus has been identified. A strict range of scores in the outcome measures should be set as inclusion criteria and the results of the trials should be clearly generalized in the relevant population. Outcome measures should be selected based on intervention and targeting related tinnitus dimensions, in line with current literature [92]. Comorbidity of hearing loss and its level should be also taken into account, not only as inclusion criterion, but also as a potential profiling factor for patients and treatment response. Tinnitus pitch, minimum masking level, and residual inhibition should also be considered as prognostic factors. Patients should be monitored for at least six months, in order to ensure stabilized results. Overall, trial designs and analysis should overcome the classic schema of comparing two interventions at two points: tinnitus is complex and heterogenous and requires identification of certain subgroups who are prone to certain treatments and of prognostic factors for treatment outcome.

## Figures and Tables

**Table 1:** Characteristics of included studies: Inclusion criteria.

Author, Year	Primary Objective	*N*	Age (Years)	Tinnitus as Primary Complaint	Tinnitus Onset (Months)	Tinnitus Laterality Uni/Bilateral	Minimum Tinnitus Threshold	Hearing Loss	Use of Hearing Aids Considered
Anders, 2010 [16]	Evaluation of the efficacy of 1 Hz repetitive transcranial magnetic stimulation (rTMS) in the treatment of tinnitus.	42	18–70	No	>6 months	Both	No	Age-adjusted normal sensorineural hearing	No
Biesinger, 2010 [17]	Effect of a Qigong intervention on patients with tinnitus with somatosensoric components	80		Yes	>3 months	Not determined	No	Normal audiogram	No
Dehkordi, 2011 [18]	Effect of gabapentin therapy on idiopathic tinnitus	80	18–85	No	>2 months	Unilateral	No	Not determined	No
Sziklai, 2011 [19]	Effect of pramipexole, a dopamine receptor agonist, influenced tinnitus associated with presbycusis	40	>50	No	>1 year	Not determined	No	Bilateral SNHL	No
Westin, 2011 [20]	Comparison of acceptance and commitment therapy (ACT) with tinnitus retraining therapy (TRT) on tinnitus	64	≥18	Yes	≥6 months	Not determined	THI ≥ 30	Hearing thresholds which would allow for the use of wearable sound generators	No
Cima, 2012 [21]	Effect of cognitive behavioral therapy (CBT) versus usual care	492	>18	No		Not determined	No	Not determined	No
Han, 2012 [22]	Comparison between Clonazepam and gingko biloba	38		No	2 months	Not determined	No	Not determined	No
Hesser, 2012 [23]	Effects on global tinnitus severity of 2 Internet-delivered psychological treatments, acceptance, and commitment therapy (ACT) and CBT, in guided self-help format	99	>18	No	>6 months	Not determined	THI ≥ 38	Not determined	No
Hoare, 2012 [24]	Comparison between different methods of frequency discrimination training on tinnitus percept	70		No	6 months	Not determined	No	<40 dB in at least one frequency	No
Jeon, 2012 [25]	Effect of acupuncture versus sham	33	18–60	No	6 months	Unilateral	No	Mean of 0.5, 1, and 2 kHz Audiogram > 50 dB	No
Kreuzer, 2012 [26]	Efficacy of a specific mindfulness- and body-psychotherapy based program in patients suffering from chronic tinnitus	36	18–80	No	>6 months	Not determined	No	Not determined	No
Ngao, 2012 [27]	Effect of transmeatal low-power laser stimulation (TLLS)	43		Yes		Not determined	No	Not determined	No
Plewnia, 2012 [28]	Safety and efficacy of bilateral CTBS to the temporal and temporoparietal cortex in the treatment of chronic tinnitus.	48		No	<5 years	Not determined	No	Not determined	No
Rocha, 2012 [29]	Efficacy of myofascial trigger point (MTP) deactivation for tinnitus relief in patients with myofascial pain syndrome	71		No	>3 months	Not determined	No	Not determined	No
Tass, 2012 [30]	Comparison between CR (4 different groups) vs. placebo	63	>18	No	6 months	Not determined	No	<50 dB in all frequencies	No
Choi, 2013 [31]	Comparison between intratympanic steroids and placebo	30		No		Not determined	No	Not determined	No
Coelho, 2013 [32]	Effect of zinc versus placebo	115	>60	No	6 months	Not determined	No	Not determined	No
Hoekstra, 2013 [33]	Effect of repetitive transcranial magnetic stimulation(rTMS) on tinnitus	50		No	>2 months	Not determined	No	Not determined	No
Mollasadeghi, 2013 [34]	Effect of low laser beam in tinnitus	89	≤50	No		Not determined	No	>15 dB at least at one of 3, 4, and 6 kHz	No
Nyenhuis, 2013 [35]	The efficacy of CBT-oriented interventions for acute tinnitus on a broader data basis.	185	18–75	No	2–26 weeks	Not determined	No	Not determined	No
Sönmez, 2013 [36]	Comparison between ozone and betahistine	68	18–75	No	6 months	Not determined	No	No	No
Taslimi, 2013 [37]	Effect of ondansedron	53	18–70	No	3 months	Not determined	No	Not determined	No
Dos Santos, 2014 [38]	Evaluation of combined use of amplification and sound generator and their combination	49		No	At least 6 months	Not determined	THI > 20	Mild to moderate symmetrical sensorineural hl	No
Hoare, 2014 [39]	Frequency discrimination training (FDT) delivered in a gaming format have significant therapeutic benefit in tinnitus	60		No		Not determined	No	≥20 dB in at least one frequency, ≤40 dB average	No
Jasper, 2014 [40]	Effects of conventional face-to-face group cognitive behavioral therapy (GCBT) and an Internet-delivered guided self-help treatment iCBT on tinnitus distress	128	≥18	Yes	≥6 months	Not determined	THI ≥ 18 or mini-TQ ≥ 8	Not determined	No
Shekhawat, 2014 [41]	Comparison of multisession anodal transcranial direct current stimulation (TDCS) of the left temporoparietal area would enhance sound therapy from hearing aids.	40		No	>2 years	Not determined	TFI > 25	Aidable HL	No
Teismann, 2014 [42]	Combine (TMNMT) with transcranial direct current stimulation (TDCS) in an effort to modulate TMNMT efficacy in the treatment of tinnitus	34		No	≥3 months	Both	No	Not determined	No
Dehkordi, 2015 [43]	Effect of low-dose laser therapy on chronic cochlear tinnitus	66		No		Not determined	No	Not mentioned	No
Bilici, 2015 [44]	5 groups: 3 types of rTMS, paroxetine, placebo	75		No	1 year	Not determined	No	Normal hearing	No
Folmer, 2015 [45]	Effect of repeated transcranial magnetic stimulation	61		No		Not determined	No	Not determined	No
Kreuzer, 2015 [46]	Comparison of medial frontal stimulation with double cone coil and conventional prefrontal left dorsolateral prefrontal cortex (DLPFC)-stimulation (study arm 2/control group) both followed by stimulation of the left temporo-parietal junction area	40		No	>6 months	Not determined	No	Not determined	No
Malinvaud, 2015 [47]	Comparison between CBT and virtual reality interactive intervention	148	18–70	No	12 months	Unilateral	No	Normal to mild	No
Pal, 2015 [48]	Investigation of the efficacy and safety of repeated sessions of a novel transcranial direct current stimulation (TDCS) protocol by combining bilateral cathodal TDCS to the auditory cortex (AC) with anodal stimulation of the prefrontal cortex (PFC).	42		No	≥1 year	Not determined	No	Age-adjusted normal hearing according to the presbycusis scale	No
Thabit, 2015 [49]	Effect of different types of rTMS and their combination	30	>18	No	6 months	Both	No	Not determined	No
Albu, 2016 [50]	Effectiveness of intratympanic (IT) steroids and melatonine versus melatonine only in acute tinnitus	60		No	Acute (within 3 months)	Unilateral	No	Not mentioned	No
Doi, 2016 [51]	Effectiveness of acupuncture therapy for tinnitus	50		No	Not determined	Not determined	THI: moderate to severe	Not determined	No
Henry, 2016 [52]	Effect on tinnitus severity by using tm-TRT-ted	148		No		Not determined	No	Not determined	No
Laureano, 2016 [53]	Effect of acupuncture on brain perfusion using (99m) ethyl cysteinate dimer single-photon emission computed tomography ((99m) Tc-ECD SPECT) in patients with tinnitus	57	18–60	No	>3 months	Both	No	Up to 25 dB	No
Lehner, 2016 [54]	Comparison between two types of rTMS	49	18–70	No	6 months	Not determined	THI > 38	Not determined	No
Li, 2016 [55]	Compare the effects of personalized, altered music to unaltered music on subjective tinnitus	34	≥18	No	≥12 months	Both	THI > 26	Hearing loss ≤70 dB	No
Lim, 2016 [56]	Efficacy of cilostazol, a selective phosphodiesterase 3 inhibitor, in patients with chronic tinnitus	50	>19	No	3–12 months	Both	Vas ≥ 4	Not determined	No
Rojas-roncancio, 2016 [57]	Effect of manganese and lipoflavonoid plus on tinnitus	40		No	>6 months	Not determined	Tinnitus loudness and annoyance > 50%	Not determined	No
Roland, 2016 [58]	Evaluation of the neural network changes in patients with bothersome chronic tinnitus who underwent rTMS treatment targeting the left temporoparietal junction (TPJ), as compared to those who received sham therapy.	30	18–60	No	≥6 months	Not determined	THI > 30	Not determined	No
Singh, 2016 [59]	Effect of B12 versus placebo	40	18–60	No	6 months	Not determined	No	Not determined	No
Stein, 2016 [60]	The effect of a sound therapy (tailor-made notched music training, TMNMT) against tinnitus	100	18–70	No	≥3 months	Both	No	Hl ≤70 dB hl in the frequency ranges of one-half octave above and below the tinnitus frequency	No
Weise, 2016 [61]	Effect of iCBT	61	>18	Yes	>6 months	Not determined	THI > 38 or mini-TQ > 13	Not determined	No
Wise, 2016 [62]	Effects of an auditory attention training game with those of a control game across tinnitus, attention, and electrophysiological measures	31	18–70	No	>6 months	Not determined	Tinnitus problem rating scale > mild	<80 Db HL nonconductive HL	No
Zarenoe, 2016 [63]	Effects of motivational interview (MI) as an adjunct to regular HA fitting for patients with tinnitus and hearing loss.	46		No		Not determined	No	Not determined	No
Elzayat, 2016 [64]	To evaluate the effectiveness of adding lidocaine to intratympanic steroid in the patients with idiopathic subjective tinnitus (IST).	44		No		Not determined	No	Not determined	No
Kallogjeri, 2017 [65]	To evaluate the effect of the brain fitting program-tinnitus on tinnitus.	60	20–65	No	>6 months	Not determined	According to bothersome scale	Not determined	No
Kim, 2017 [66]	Effect of different approaches of acupuncture	39	20–75	No	2 weeks	Not determined	No	Not determined	No
Landgrebe, 2017 [67]	Evaluation of the efficacy of a two-week 1-Hz-RTMS in patients with chronic tinnitus.	146	18–70	No	>6 months	Not determined	THI > 38	Normal, age-adjusted hearing levels. Conductive hearing loss ≤ 15 db.	No
Mckenna, 2017 [68]	Effect of mindfulness based cognitive therapy (MBCT) in tinnitus severity, psychological distress, functional disability, avoidance, and negative cognitions and a greater increase in tinnitus acceptance.	75	≥18	No	>6 months	Not determined	No	Hearing levels allowing participation in group discussions	No
Arif, 2017 [69]	Relaxation therapy and mindfulness	61	>18	Yes		Not determined	No	Not determined	No
Beukes, 2017 [70]	Efficacy of guided internet based cognitive behavioral treatment (iCBT)	146	>18	No	>3 months	Both	TFI > 25	Not determined	No
Sahlsten, 2017 [71]	E-field navigation should versus non-navigated rTMS	39	18–65	No	6 months–10 years	Both	No	Not determined	No
Theodoroff, 2017 [72]	To determine if an acoustic stimulus mimicking the tinnitus perception delivered during sleep from the Otoharmonics corporation’s LEVO system reduces tinnitus-related distress and/or perceived loudness of tinnitus during awake hours for people who experience bothersome tinnitus	58	30–72	No	>6 months	Not determined	TFI > 25	<70 dB hl, in all frequencies between 0.25 and 8 kHz	No
Tyler, 2017 [73]	Effect of vagus nerve stimulation (VNS) paired with sounds in chronic tinnitus patients	30	22–65	No	>1 year	Both	No	Not determined	No
Lee, 2018 [74]	Effect of intratympanic steroids on acute tinnitus	54		No	Acute (one month)	Unilateral	No	Not determined	No
Beukes, 2018 [75]	Evaluation of an Internet-based cognitive behavioral therapy intervention versus face to face	92	>18	No	Not determined	Not determined	No	Not determined	Yes
Abtahi, 2018 [76]	Effectiveness of anodal and cathodal methods in reducing the intensity of tinnitus	51	18–80	No	>1 year	Not determined	No	Not determined	No
El Beaino, 2018 [77]	Effect of sulodexide (heparin and dermatan) vs. placebo	124	>18	No	12 months	Not determined	No	Not determined	No
Hong, 2018 [78]	Effect of nitrous oxide on tinnitus		18–65	No	>6 months	Not determined	According to bothersome scale	Not determined	No
Godbehere, 2019 [79]	Theta burst TMS are an effective treatment for chronic tinnitus	40	>18	No	Not determined	Both	No	No HL, mild and moderate HL	No
Hall, 2019 [80]	Effect of AUTt00063, a novel centrally acting drug) potent and selective modulator of kv3.1 and kv3.2 voltage-gated potassium channels) vs. placebo	76	>18	Yes	>6, <18 months	Both	TFI > 24 and <68	<60 db in 0.5,1,2,4 kHz	No
Li, 2019 [81]	Clinical efficacy of cognitive behavioral therapy (CBT) for treatment of chronic subjective tinnitus	100		No	>3 months	Not determined	No	Not determined	No
Noh, 2019 [82]	To investigate the effects of active dual-site rTMS treatment on reducing tinnitus using a double-blind randomized controlled trial.	30		No		Not determined	No	Not determined	No
Prozchazkova, 2019 [83]	Comparison between gingko biloba and pentoxifylline	197	>30	No	3 months	Not determined	Mini TQ > 5	Not determined	No
Radunz, 2019 [84]	Comparison between ginkgo biloba, HA, and their combination	35	>18	No	3 months	Both	No	All types of hearing loss	No
Sahlsten, 2019 [85]	Comparison of neuronavigated versus non-navigated repetitive transcranial magnetic stimulation	40	18–65	No	6 months–10 years	Both	Numeric scale > 4	Not determined	No
Scherer, 2019 [86]	To compare the efficacy of tinnitus retraining therapy (TRT) and its components, ST, and TC, with the standard of care (SOC) in reducing the negative effect of tinnitus on quality of life.	98		No	>1 year	Not determined	TQ > 40	Functionally adequate hearing sensitivity without requirement of amplification	No
Yakunina, 2019 [87]	Evaluation of the effects on tinnitus of hearing aids (HA) alone without accompanying counseling or any other therapy additionally, whether FL techniques (LFT and FT) performed compared with conventional WDRC in the same open-fit HA in terms of tinnitus suppression for patients with high frequency hearing loss (HFHL).	94	>18	No	≥3 months	Not determined	THI > 18 Vas ≥ 50%	SNHL	No
Tutar, 2020 [88]	Efficacy of transcutaneous electric stimulation applied to the auricula	60	18–65	No	>3 months	Not determined	No	Not determined	No

**Table 2:** Characteristics of included studies: study procedures and outcome measures.

Author, Year	Treatment	Control Group Intervention	Randomization	Outcome Measures	Monitoring Duration	Power Analysis	Results	Is Treatment Effective?
Abtahi, 2018 [76]	Anodal Stimulation, Cathodal Stimulation	Sham Stimulation	Unclear	Tinnitus Intensity Variations on A Scale Between −4 and +4. In This Scale, −4 Indicated Worsening Conditions, +4 Meant Full Recovery, And Zero Conveyed No Change in The Tinnitus Intensity.	2 Months	No	Anodal Stimulation Was More Effective Than the Cathodal and Control Stimulation in Reducing the Intensity of Tinnitus in The Short Term	Yes, Between Two Versions of The Same Treatment
Albu, 2016 [50]	Intratympanic (IT) Steroid and Melatonin	Melatonin	Unclear	THI, PSQI, BDI	3 Months	No	Better Response in The Combined Group of Melatonine And IT In Acute Tinnitus Patients	Yes
Anders, 2010 [16]	Active or Sham repetitive transcranial magnetic stimulation (rTMS)	Sham Rtms	Unclear	VAS, THI	26 Weeks	No	1 Hz Rtms Treatment Was Capable of Significantly Reducing the Total Baseline Score of Basic Scales That Measure Tinnitus Severity	No
Arif, 2017 [69]	Relaxation Therapy or Mindfulness Meditation Treatment Over A Period Of 15 Weeks	Relaxation Procedure	Clear	Primary: TRQ Secondary VAS and A Health State Thermometer.	15 Weeks	No	Changes in Tinnitus Loudness and THI (but not TRQ) with the Customized Sound Therapy Were Statistically Greater Than Those of The Broadband Noise Therapy	No
Beukes, 2017 [70]	Internet-based cognitive behavioral treatment (iCBT) Intervention	ICBT After 8 Weeks	Algorithm Implemented by Independent Researcher	Primary: TFI, Secondary: ISI, GAD-7, PHQ-9, HHIA-S, HQ, CFQ, SWLS	2 Months	80%	Guided ICBT For Tinnitus Using Audiological Support Resulted in Statistically Significant Reductions in Tinnitus Distress and Comorbidities (Insomnia, Depression, Hyperacusis, Cognitive Failures) And Improved Quality of Life.	Yes
Beukes, 2018 [75]	Internet- Based Intervention	Face-To-Face Tinnitus Care	Unclear	THI, TFI	2 Months	90%	ICBT And F2F Interventions Are not Effective for Reducing Tinnitus Distress and Most Tinnitus-Related Difficulties.	No
Biesinger, 2010 [17]	10 Qigong Training Sessions	No Treatment	Unclear	VAS, TBF-12	3 Months	No	No Statistically Significant Changes in Both Groups	No
Bilici, 2013 [44]	rTMS	Paroxetine, Placebo	Unclear	THI, TSI, BAS, PSS	6 Months	No	No Significant Improvement Neither for Rtms Groups nor For Controls	No
Choi, 2013 [31]	IT Steroids	Placebo	Clear	THI. VAS	1 Month	No	No Significant Difference Between IT Steroids and Placebo	No
Cima, 2012 [21]	CBT	Usual Care	Clear	HUI, HADS, TFQ	12 Months	No	Superiority Of CBT	Yes
Coelho, 2013 [32]	Zinc	Placebo	Unclear	THQ	4 Months	90%	No Significant Differences Between Zinc and Placebo	No
Dehkordi, 2011 [18]	Gabapentin	Placebo	Unclear	TSI	26 Months	No	No Statistically Significant Difference Between the Two Groups In TSI.	No
Dehkordi, 2015 [43]	Active Laser Treatment	Inactive Dummy Treatment	Unclear	TSI	4 Weeks	No	No Statistically Significant Improvement Neither in Laser nor In Control Group	No
Doi, 2016 [51]	Acupuncture	No Treatment	Randomization Was Carried Out with The Aid of Computerized Table of Random Numbers Created by A Microsoft Excel Spreadsheet.	VAS, THI	5 Weeks	No	Treatment with Acupuncture Improves the Perception of Tinnitus, Decreases the Intensity Level, Hence There Is No Comparison Between Levels of Improvement	Yes, Against Placebo In 5 Weeks, However No Comparison of Decrease
Dos Santos, 2014 [38]	Hearing Aids + Sound Generator	Hearing Aids	Unclear	THI	3 Months	80,0%	No Superiority of The Combined Use of Amplification and Sound Generator Over Conventional Amplification Alone in Reducing the Discomfort of Tinnitus. Both Groups Presented Similar Responses in Both Reduction of Discomfort Caused by Tinnitus	No
El Beaino, 2018 [77]	Sulodexide	Placebo	Unclear	THI, Mini TQ	Right After Treatment	80%	Improvement in THI and Mini TQ Right After the End of Treatment with Sulodexide	Yes
Elzayat, 2018 [64]	Group A Was Injected with Combined Lidocaine 2% And Dexamethasone 8 Mg/2 mL (ITLD). Group B Was Injected Only by Dexamethasone 8 Mg/2 ML. (ITD).	ITD As A Controlled Group	Clear	THI, VAS, ATQ	6 Months	No	Both Treatments Were Effective but No Difference Between Groups Was Found	Yes
Folmer, 2015 [45]	rTMS Daily For 2 Weeks	Sham Rtms With A Same Looking Coil	Unclear	TFI	26 Months	No	Significant Improvement in Active Compared to Placebo Group	Yes
Godbehere, 2019 [79]	Theta Burst TMS	Placebo Arm	Unclear	TFI	4 Weeks	No	No Significant Difference in Scores Between the Active Treatment Group and The Sham Control Group	No
Hall, 2019 [80]	AUT00063	Placebo	Clear	TFI, VAS	28 Days	90%	No Significant Improvement for Both Groups (Channel Blocker and Placebo)	No
Han, 2012 [22]	Clonazepam	Ginkgo Biloba	Unclear	THI, VAS, Loudness Scale		No	Improvement with Use of Clonazepam and Not Gingko Biloba, but Right After Treatment	Yes
Henry, 2016 [52]	TM-TRT-TED	No Treatment	Clear	THI	18 Months	80%	No Statistically Significant Improvement In THI. By 6 Months, The TED Group Showed Significant Improvement from Baseline and Its Improvement Was Not Significantly Different from That Shown in TM Or TRT.	No
Hesser, 2012 [23]	CBT Or ACT	Monitored Internet Discussion Forum	Clear	Primary: THI, Secondary: HADS	1 Year	80%	The Effect of ACT Compared with The Control Condition at Posttreatment on The Primary Outcome Was in The Moderate Range and Comparable to The Effect Observed Following CBT (D = 0.68 vs. D = 0.70).	No
Hoare, 2012 [24]	Frequency Training	Different Frequency Training		THQ	4 Weeks	80%	Statistically and Clinically Meaningful Improvement in All Groups. No Difference Between Groups	Yes
Hoare, 2014 [39]	To Play A Tailored Video Game For 30 Minutes, 5 Days A Week For 4 Weeks	Another Type Of FDT	Clear	THQ	4 Weeks	80%	Statistically but Not Clinically Significant Changes in One of The Games Used	No
Hoekstra, 2013 [33]	rTMS in 1000Hz	Placebo	Unclear	Primary: TQ. Secondary THI, VAS	6 Months	80%	No Significant Difference Between Groups	No
Hong, 2018 [78]	40 Minutes Session of Nitrous Oxide Under General Anesthesia	Same Procedure Without Nitrous Oxide	Clear	TFI	2 Weeks	81%	No Significant Differences Between Intervention and Control Group. Neither Groups Had Clinical or Statistically Significant Improvement	No
Jasper, 2014 [40]	GCBT, iCBT	Web-Based Discussion Forum (DF)	Unclear	THI, Mini-TQ, Secondary: HADS, ISI, TAQ	6 Months	No	ICBT And Conventional GCBT Do Not Have Significant Differences Effects on Tinnitus Distress and Associated Problems.	No
Jeon, 2012 [25]	Acupuncture	Sham	Unclear	THI, VAS		No	No Significant Differences Between Acupuncture and Sham	No
Kallogjeri, 2017 [65]	Brain fitness program tinnitus (BFP-T)	No Treatment	Unclear	THI, TFI, Global Bother Score	8 Weeks	85%	No Statistically Significant Changes Between Study Groups.	No
Kim, 2017 [66]	Manual Acupuncture	Electroacupuncture	Unclear	THI, VAS		80%	No Significant Improvement for Any Acupuncture Group In Regards To THI and Loudness	No
Kreuzer, 2012 [26]	Mindfulness and Body Group Therapy	Waiting List (Therapy After 24 Weeks)	Unclear	TQ	24 Weeks	No	A Significant Reduction in The TQ Score (Baseline vs. Week 9) Compared to The Waiting List Control Group, However Difference Was Not Stable in Long Term F/U	No
Kreuzer, 2015 [46]	Medial Frontal Stimulation with Double Cone Coil + Stimulation of The Left Temporo-Parietal Junction Area	Conventional Prefrontal Left DLPFC-Stimulation + Stimulation of The Left Temporo-Parietal Junction Area	Unclear	TQ, Secondary: THI, CGI-CHANGE, Whoqol-Bref-Questionnaire	12 Weeks	No	ΤICDC-Stimulation Non-Superior to Standard Rtms Regarding Both Primary and Secondary Outcome Measures.	No
Landgrebe, 2017 [67]	2 Week Treatment Real 1-Hz-Rtms vs. Sham Rtms	Sham Rtms	Clear	Primary: The Change of Tinnitus Severity Assessed by Means of The Change of The TQ Sum Score Between Baseline Score vs. Day 12. Secondary: Changes of The TQ Sum Score, The THI and TSS During the Treatment and The Follow-Up Period. Further: Changes of Overall Illness Severity, Changes in Depressive Symptoms, Changes in Quality of Life and Changes in Psychoacoustic Measures of Tinnitus.	26 Weeks	No	Real 1-Hz-Rtms Applied to The Left Temporal Cortex Did Not Provide Any Therapeutic Benefit as Compared to Sham Treatment in Patients with Chronic Tinnitus.	No
Laureano, 2016 [53]	True Acupuncture 99mTC-ECD SPECT	Sham Acupuncture	Unclear	Primary: SPECT Measurements, Secondary: THI, VAS, HAS, BDI	12 Weeks	80%	No Significant Differences After Treatment Were Observed with Regard to the VAS, HAS or BDI Between the Treatment Groups.	No
Lee, 2018 [74]	IT Steroids	Placebo (Saline)	Clear	THI, VAS	1 Month	80%	No Difference Between IT And Placebo Groups	No
Lehner, 2016 [54]	High Frequency rTMS	Single Site Rtms	Clear	TQ, THI,	6 Months	80%	No Difference Between Groups	No
Li, 2016 [55]	Music Altered by The Software to Treat Tinnitus	Unaltered Music	Unclear	THI, TFI, HADS	12 Months	80%	Statistically Significant and Clinically Meaningful Effects of The Therapy as Indicated by The Consistent Treatment-Control Group Difference in THI Score and The Significant Reduction in THI Score Within the Treatment Group During The 12-Month Period.	Yes
Li, 2019 [81]	Masking Therapy+ Sound Treatment + CBT	Masking Therapy + Sound Treatment (Tinni Test)	Unclear	THI, SCL-90	6 Months	No	Effective Rate in Intervention Group Was Significantly Higher Than That in Control Group (P < 0.01)	Yes
Lim, 2016 [56]	Oral 100 mg Cilostazol	Placebo	Unclear	VAS, THI, SF-36	4 Weeks	No	THI Failed to Show A Significant Drug Effect of Cilostazol	No
Malinvaud, 2015 [47]	Virtual reality (VR)	CBT	Clear	STSS, THI, THQ, VAS		No	Both VR And BT Groups Improved	Yes
Mckenna, 2017 [68]	RT Or MBCT Treatment vs. Waiting Period Without Treatment	RT Treatment Group	Clear	Primary: TQ, CORE-NR, Secondary: CORE-OM, VAS, TFI, HADS, TCS, T-FAS, TAQ, MAAS, WSAS	6 Months	80.0%	MBCT Is More Effective in Reducing Tinnitus Severity Than Both A Waiting Period and An Active Treatment of Equal Intensity (RT)	Yes
Mollasadeh, 2013 [34]	Low Laser Beam	Placebo	Unclear	THI, VAS, Loudness Scale	3 Months	No	Larger Improvement in Low Beam Laser Compared to Placebo, Hence More Than 50% Of Intervention Group Without Improvement	Yes
Ngao, 2012 [27]	TLLS	Sham	Unclear	THI, VAS	Right After Intervention	No	No Significant Difference Between TLLS And Sham	No
Noh, 2019 [82]	Dual-Site Rtms Or Sham Rtms	Sham Rtms	Unclear	Primary: THI, Secondary: VAS	8 Weeks	No	A Beneficial Effect of Rtms On Tinnitus Suppression Was Found in The Dual-Site Active Rtms Group, but Not in The Sham Rtms Group.	Yes
Nyenhuis, 2013 [35]	Internet Training, Bibliotherapy, Group Treatment or An Information-Only Condition.	Information Only	Clear	Primary: TQ, Secondary: BL, PHQ-D	9 Months	80.0%	Improvement Rates Were Higher in The Active Training Conditions Than in The Control Condition, And Deterioration Rates Were Generally Lower in The Training Conditions In TQ.	Yes
Pal, 2015 [48]	transcranial direct current stimulation (TDCS)		Unclear	Primary: THI, Secondary: STSS, HAD, VAS, CGI	3 Months	80.0%	This TDCS Protocol Did Not Show A Beneficial Effect on Tinnitus.	No
Plewnia, 2012 [28]	CTBS Over the Secondary Auditory Cortex (SAC), The Temporoparietal Association Cortex (TAC), Or Sham Stimulation [Placebo (PLC)].	Placebo	Clear	TQ	3 Months	80%	No Difference Between Real and Sham Treatments nor Between Temporal and Temporoparietal Ctbs.	No
Prozchazkova, 2019 [83]	Ginkgo Biloba	Pentoxifylline	Unclear	VAS, Mini TQ, HADS	3 Months	No	Both Gingko Biloba and Pentoxifylline Improve Mini TQ. No Difference Between Groups	Yes
Radunz, 2019 [84]	Gingko Biloba	HA	Unclear	THI, VAS	6 Months	No	Both Gingko Biloba Improved Compared to Baseline, No Difference Between Groups Though, Apart from Long Lasting Tinnitus	Yes
Rocha, 2012 [29]	10 Sessions of Myofascial Trigger Point Deactivation	10 Sessions with Sham Deactivation	Unclear	THI	3 Months	No	MTP Deactivation Through Digital Pressure Was Deemed Effective in Each and Every Tinnitus Variable Under Evaluation and In the Medium Run Responsiveness to Treatment Remained Stable In 75.8% Patients.	Yes
Rojas-Roncancio, 2016 [57]	Manganese and Lipoflavonoid Plus	Lipoflavonoid Plus	Unclear	THQ, TPFQ	6 Months	No	No Significant Improvement in Both Groups	No
Roland, 2016 [58]	Sham or Active Treatment rTMS to TPJ	Sham Rtms to TPJ	Clear		2 or 4 Weeks	No	No Changes in Neural Connectivity Following Rtms Therapy. Results Suggest Instead That the TPJ May Not Be an Ideal Target for Tinnitus Treatment.	No
Sahlsten, 2017 [71]	rTMS	Placebo Rtms	Unclear	THI, VAS	6 Months	80%	Improvement for The VAS Scores (Intensity, Annoyance, Distress) And THI Scores Both in The Active Rtms Group and The Placebo Group.	No
Sahlsten, 2019 [85]	rTMS With and Without Neuronavigation for 2 Weeks In 2000 Hz	With and Without Neurostimulation	Unclear	THI, Global Impression of Change	3 Months	No	No Significant Difference Between Groups, However Significant Improvement in Both	No
Scherer, 2019 [86]	TRT, including tinnitus specific counseling (TC) and sound-therapy (ST) Implemented with Ear- Level sound generators (SGS); Partial TRT, Including TC and Placebo SGS; Or Standard of Care (SoC)	Placebo SGS Or Standard of Care (Soc)	Clear	Primary: TQ, Secondary: TFI, THI, VAS	18 Months	80.0%	No Meaningful Differences Between TRT And Soc (Our Primary Comparison) Or Between Partial TRT And Soc Or TRT (Our Secondary Comparisons).	No
Shekhawat, 2014 [41]	Transcranial direct current stimulation (TDCS)	Sham Stimulation	Clear	TFI, THI, THQ, MML	7 Months	No	No Significant Differences for Any of The Questionnaires; Decrease in MML For the RTMS Group	No
Singh, 2016 [59]	B12	Placebo	Unclear	Matching, TSI	1 Month	No	Improvement in Patients with B12 Insufficiency, Hence in A Very Small Sample	No
Sönmez, 2013 [36]	Ozone	Betahistine	Unclear	THI, Loudness Scale	6 Months	No	No Differences Between Ozone and Betahistine, Both Showed Improvement Compared to Baseline Though	Yes
Stein, 2016 [60]	Fixed Notch-TMNMT	Placebo (Moving Notch)	Clear	THQ, VAS	4 Months	90.0%	Tinnitus Loudness and Other Measures of Tinnitus Distress Do Not Show Relevant Changes.	No
Sziklai, 2011 [19]	Pramipexole	Placebo	Clear	THI	4 Weeks	No	No Cumulative Analysis. Greater Proportion of Patients Reporting Tinnitus Disappearance in The Interventional Group. Results Not Confirmed by Electrocochleography.	Unclear
Taslimi, 2013 [37]	Ondansedron	Placebo	Clear	THI, TSI, VAS, HADS		No	No Significant Differences Between Ondansedron And Placebo	No
Tass, 2012 [30]	CR	Placebo		VAS, TQ	12 Weeks	No	Improvement Before and After Treatment and Also Compared to Placebo	Yes
Teismann, 2014 [42]	Anodal TDCS, Cathodal TDCS + TMNMT	Sham Stimulation + TMNMT	Unclear	THQ, THI, TQ	31 Days	No	No Significant Modulating Effect of TDCS Polarity: Significant Main Effects or Interactions of TDCS Condition Were Neither Found in The Primary Outcome Measure nor In Any of The Secondary Outcome Measures (THI, TQ, Or Loudness VAS;	No
Thabit, 2015 [49]	rTMS	RTMS	Unclear	THI, VAS	1 Month	No	Combination Treatment Significantly Better	Yes
Theodoroff, 2017 [72]	LEVO System with A Tinnitus-Matched Stimulus (TM Group) vs. LEVO System with A Noise Stimulus (NS Group; White Noise And/or Band Noise) vs. Marsona 1288 Sound Conditioner/Tinnitus Masker (Bedside Sound Generator Device; BSG Group).	BSG And NS Groups, but Not in The Same Manner as A Placebo-Controlled Group	Unclear	TFI, NRS, And LM at 1 kHz	3 Months	No	Greater Average Improvement in Reactions to Tinnitus with TM or NS Devices Compared to The BSG Device.	Yes
Tutar, 2020 [88]	10 Sessions Of 30 Minutes in One Month	Placebo	Unclear	THI, DASS	4 Weeks	No	Significant Improvement in Uni- And Bilateral Groups Compared to Placebo	Yes
Tyler, 2017 [73]	VNS Implant-Paired	VNS Implant-Unpaired (Paired After 6 Weeks)	Clear	THI, TFI, THQ	1 Year	No	No Significant Differences for Any of The Outcome Measures	No
Weise, 2016 [61]	10-Week Guided Internet-Based Self-Help Program	DF	Unclear	Primary: THI, Mini-TQ, Secondary: HADS	1 Year	No	ICBT Resulted to Significantly Better THI Scores Compared to Participation in An Online Forum	Yes
Westin, 2011 [20]	ACT, TRT	Wait List Control	Unclear	Primary: THI, Secondary: ISI, QOLI, HADS, CGI-I	18 Months	80%	ACT Is More Effective in Reducing Tinnitus Impact Than Tinnitus Retraining Therapy or Being on A Wait List.	Yes
Wise, 2016 [62]	Experimental Attention Training Game (“Terrain”)	A Control Game (“Tetris”)	Unclear	TFI, Secondary: THI, Tinnitus Severity Numeric Scales	20 Days	92%	TFI Scores Improved Following The 20-Day Use for the “Terrain” Program Compared with The Nonauditory “Tetris” Group.	Yes
Yakunina, 2019 [87]	HAs With WDRC, HAs With FT, Or HAs With LFT	FL Techniques (LFT and FT) Group	Clear	Primary: THI, Secondary: VAS	6 Months	80.0%	No Significant Differences Were Found Between Conventional Has And FL-Type Has in Terms of Tinnitus Relief Among Patients With HFHL.	No
Zarenoe, 2016 [63]	MI Group Received A Brief MI Program, Whereas Patients in The SP Group Underwent Conventional Hearing Aid Fitting.	Conventional Hearing Aid Fitting Group	Unclear	THI, IOI-HA	3 Months	No	The MI Intervention Did Not Appear to Have Any Additional Effect on Hearing Aid Fitting Compared to Conventional Hearing Rehabilitation.	No
